# High proportion of anergic B cells in the bone marrow defined phenotypically by CD21(−/low)/CD38- expression predicts poor survival in diffuse large B cell lymphoma

**DOI:** 10.1186/s12885-020-07525-6

**Published:** 2020-11-03

**Authors:** Sewa Rijal, Johanna Kok, Caitlin Coombes, Lillian Smyth, Jayde Hourigan, Sanjiv Jain, Dipti Talaulikar

**Affiliations:** 1 Australian National University Medical School, College of Medicine, Biology and Environment, Canberra, Australia; 2 Haematology Translational Research Unit, Department of Hematology, Canberra Hospital, Canberra, Australia; 3 Department of Diagnostic Genomics, Canberra Hospital, Canberra, Australia; 4 Department of Anatomical Pathology, Canberra Hospital, Canberra, Australia

**Keywords:** Diffuse large B cell lymphoma, B cells, Prognosis, Microenvironment, Anergic

## Abstract

**Background:**

Diffuse large B cell lymphoma (DLBCL) is the commonest lymphoma that is highly aggressive where one-third of the patients relapse despite effective treatment. Interaction between the lymphoma cells and the non-clonal immune cells within the bone marrow microenvironment is thought to play a critical role in the pathogenesis of DLBCL.

**Methods:**

We used flow cytometry to characterize the proportion of B cell subpopulations in the bone marrow (*N* = 47) and peripheral blood (*N* = 54) of 75 DLBCL patients at diagnosis and study their impact on survival.

**Results:**

Anergic B cells in the bone marrow (BM), characterized as having CD21(−/low)/CD38- expression, influenced survival with high numbers (defined as > 13.9%) being associated with significantly shorter overall survival (59.7 months vs 113.6 months, *p* = 0.0038). Interestingly, low numbers of anergic B cells in the BM (defined as ≤13.9%) was associated with germinal center B cell type of DLBCL (*p* = 0.0354) that is known to have superior rates of survival when compared to activated B cell type. Finally, Cox regression analysis in our cohort of patients established that the inferior prognosis of having high numbers of anergic B cells in the bone marrow was independent of the established Revised International Prognostic Index (R-IPI) score.

**Conclusions:**

High proportion of anergic B cells in the BM characterized by CD21(−/low)/CD38- expression predicts poor survival outcomes in DLBCL.

## Background

Diffuse large B cell lymphoma (DLBCL) is an aggressive lymphoma compromising of various subtypes that differ markedly in terms of morphology, immunophenotypic profile, genetics, pathogenesis and clinical outcomes [1, 2]. It is the most common subtype of non-Hodgkin lymphoma, making up about 25% of these cases [3, 4]. It is derived from the clonal expansion of abnormal B cells arising from the germinal center B cells (GCB) or post germinal center activated B cells (ABC) and primarily affects lymphoid tissues but can also be extranodal [5, 6]. Standard treatment involves chemotherapy with the addition of Rituximab, a monoclonal antibody against CD20, known as R-CHOP therapy, that has resulted in a significant improvement in overall survival (OS) [7, 8]. However, one-third of the patients have refractory disease that does not respond to treatment, or relapses with poor outcomes [9].

The revised International Prognostic Index (R-IPI) is the primary prognostic tool used to define long term survival in DLBCL patients when treated with standard therapy [10–12]. This incorporates age, performance status, serum lactate dehydrogenase levels, disease stage, and degree of extranodal involvement to calculate a score that predicts OS [10].

Emerging evidence suggests that immune cells in the tumour microenvironment (TME) that interact with the lymphoma cells play a critical role in the pathogenesis and progression of DLBCL [13, 14]. Thus, understanding the pattern of immune cell populations in DLBCL may not just help predict disease outcomes but may also have implications on treatment with immunotherapy agents.

Some studies have assessed the role of immune cell populations in the peripheral blood (PB) of DLBCL patients to define prognosis. We have previously published data to show that low lymphocyte count is significantly associated with adverse outcomes [15]. Similar findings and other parameters of high monocytes and decreased lymphocyte/monocyte ratio have also been found to be associated with poor prognosis [16–20]. Low levels of CD4+ T cells appear to be an independent predictor of inferior survival [21]. High CD8 T cells are reported to be unfavorable and high CD4:CD8 ratio is associated with improved survival [22, 23]. High numbers of regulatory T cells have also been associated with poor prognosis [24–27]. However, research on B cell populations in DLBCL microenvironment has been limited. A recent study has reported that B cells identified as CD19+ with high side scatter by flow cytometry predicts inferior survival [28].

Our study aims to look at the proportions of B cell subpopulations in the PB of DLBCL patients using multicolor flow cytometry analysis to evaluate their association with survival. We have also extended our analysis to bone marrow (BM) cells as BM involvement in DLBCL has been reported to predict clinical outcomes in some studies [29–32]. Moreover, obtainment of BM samples is a common diagnostic step in DLBCL, so cells are readily available for flow analysis. This is the first study that looks at a comprehensive panel of B cell populations in the BM and PB of DLBCL patients to define their impact on survival.

## Methods

### Patients

Seventy-five DLBCL patients who were diagnosed at The Canberra Hospital from 2002 to 2014 and had pre-treatment samples archived in the ACT Hematology Research Tissue Bank were included in the study with BM (*n* = 47) and PB (*n* = 54) samples used for flow cytometry analysis where 28/75 patients had both BM and PB samples available. PB and BM mononuclear cells (MNCs) were isolated using Ficoll-Plaque® (GE Healthcare, USA) via gradient centrifugation method at the time of diagnosis. The layer containing the MNCs was carefully removed and subjected to red cell lysis buffer treatment. 2–10 × 10^6^ total MNCs were cryopreserved in freezing medium (90% fetal bovine serum (FBS) containing 10% DMSO) and stored at − 80 °C until analysis was done.

Patient clinical data was retrospectively collected after approval from the ACT Health Human Research Ethics Committee with the approval number ETHLR.12.170 on 23/11/2011. The mean age of the 75 patients was 64 years (range 35–94 years) with more males than females (males: 41; females: 34). R-IPI data was available for all patients with R-IPI of 0 = 7 (9.3%), 1 = 11 (14.7%), 2 = 19 (25.3%), 3 = 20 (26.7%), 4 = 10 (13.3%) and 5 = 8 (10.7%). Chemotherapy that included R-CHOP (*N* = 54) or R-CHOP like therapy (*N* = 15) with maintenance rituximab was given to 69/75 patients. Treatment response in this group was available for 64/69 patient where *N* = 44 had complete response (CR), *N* = 18 had partial response (PR) and *N* = 2 died prior to completion of treatment. Other treatments included radiotherapy (N = 4) where N = 2 had CR and N = 2 had disease progression. 1 patient had PR with rituximab only therapy while 1 patient did not receive any treatment. Outcome data was available on all 75 patients with median OS of 37 months over a median follow up of 35 months (range 1–117 months). Cell of origin data in terms of ABC and GCB phenotype was derived on all samples using the Hans algorithm.

### Flow cytometry

Cells were briefly thawed in a 37 °C water bath after which pre-warmed Ca^2+^, Mg^2+^ free Hank’s Balanced Salt Solution (HBSS) (Invitrogen, USA) containing 10% FBS was immediately added to dilute the freezing medium. The cell suspension was centrifuged at 300 g for 5 mins and resuspended in HBSS containing 5% FBS. A total of 100,000–500,000 cells were stained with a cocktail of antibodies to detect B cell surface antigens and incubated in the dark for 30 mins at 4 °C. All antibodies were purchased from BD Biosciences, USA. Cells were washed twice and resuspended in a final volume of 100 μl HBSS containing 5% FBS. Cells were then applied to flow cytometry using a BD LSRFortessa cell analyser (Becton Dickinson, USA) located at The Microscopy and Cytometry Research Facility at the John Curtin School of Medical Research, Australian National University. A minimum of 50,000 and an average of 120,000 total events were collected for analysis. All flow cytometry analysis was done using Flowjo® software version 10. The cocktail of antibodies used to look at B cells and subpopulations and the fluorescence channel used are summarized below.

**Table:** 

Flourescent tag	FITC	PE	APC Cy7	Texas Red PE	PE Cy5	PE Cy 7	Alexa Fluor 405	Qdot 605	APC	Alexa Fluor 700
**B cell markers**	CD20	Lambda	Kappa	CD34	CD19	CD10	CD38	CD27	CD21	CD5

### Data analysis

**Fig. 1:**
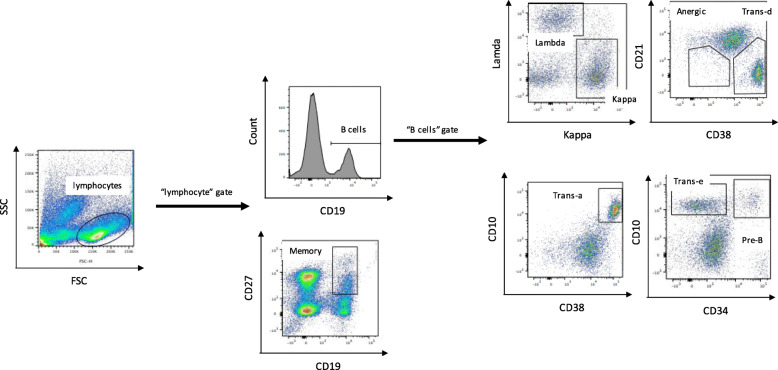
Flow cytometry gating strategy used to quantify proportions of B cells and its subpopulations. 75 DLBCL patients with BM (N = 47) and PB (N = 54) samples at diagnosis were subjected to multicolor flow cytometry to look at proportions of B cells and its subpopulations. A plot of forward versus side scatter was used to gate cells falling under the “lymphocytes” region. From the “lymphocyte” gate, B cells were gated as CD19+ and memory B cells were gated as CD19+/CD27+. Following subpopulations were further characterized on the B cell gate, i.e. on the CD19+ gate. Three categories of transitional (trans) B cells were identified as CD10+/CD34-, CD38+/CD21- and CD10+/CD38+ and labelled trans-e, trans-d and trans-a respectively. Precursor (pre-B) cells were gated as CD10+/CD34+ and anergic B cells were gated as CD21(−/low)/CD38-. Specific stains were used to identify the two types of immunoglobulin light chain in B cells, i.e. lambda and kappa populations

Flowjo® software version 10 was used to perform analysis of the flow cytometry data to identify B cells subtypes distinguished on the basis of their maturation process [33]. First, a plot of forward versus side scatter was used to gate cells falling under the “lymphocyte” region. From the “lymphocyte” gate, B cells were gated as CD19+ cells, and memory B cells were gated as CD19+/CD27+. Following subpopulations were further characterized on the B cell gate, i.e. on the CD19+ gate. Three categories of transitional B cells were identified as CD10+/CD34-, CD38+/CD21- and CD10+/CD38+ based on the varied literature and arbitrarily labelled Trans-e, Trans-d and Trans-a respectively [34–36]. They were summed up in the statistical analysis stage and treated as a single entity of transitional B cells. Precursor B cells were gated as CD10+/CD34+ and an additional sub-population of B cells, designated anergic, were gated as CD21(−/low)/CD38- [37–39]. Specific stains were used to identify the two types of immunoglobulin light chain in B cells, i.e. lambda and kappa populations. Gating strategy to quantify the proportions of B cells and subpopulations is shown in (Fig. 1).

### Statistical analysis

All statistical analysis was done using SPSS software version 11.0. Analysis comparing the proportions of B cells and subtypes at 5-year survival outcome was done using exact non-parametric Mann-Whitney U test, suitable for data that does not follow a normal distribution. For this analysis, samples with follow-up data of less than 5 years were excluded. OS survival was determined as the number of months from the date of diagnosis to the date of death or date of last follow-up (censored) and evaluated based on a Kaplan Meier analysis. The Cox proportional hazard model was used to evaluate the association between OS and experimental covariates along with known prognostic marker R-IPI. For all analysis, *p*-value of <.05 was taken to be significant unless specified.

## Results

### High proportion of anergic B cells in the BM lead to poor survival outcomes independent of marrow involvement

B cells were quantified as proportion of “lymphocyte” population that was CD19+ with further characterization of various B cell subpopulations as shown in (Fig. 1) via multicolor flow cytometry. Table 1 summarizes this data for 47 BM samples and 54 PB samples.

**Table 1:** Proportion of B cells and its subpopulations (%) as detected by multicolor flow cytometry in 75 DLBCL patients bone marrow (*N* = 47) and peripheral blood (*N* = 54) samples at the time of diagnosis

			Bone marrow					Peripheral blood				
Gated on	Cell population	Phenotype	N	Mean	SD	Min.	Max.	N	Mean	SD	Min.	Max.
%lymphocytes	B cells	CD19+	47	13.35	7.49	0.30	32.50	54	14.11	11.71	1.79	60.00
%lymphocytes	Memory	CD19+ CD27+		3.60	4.71	0.04	23.40		3.29	3.83	0.00	22.70
%B cells	lambda	lambda+		27.27	14.47	0.52	84.90		34.65	16.25	0.82	93.30
	kappa	kappa+		42.77	17.78	1.13	76.10		47.24	16.47	0.56	74.70
	Precursor	CD34+ CD10+		1.40	2.34	0.00	11.00		0.41	1.05	0.00	6.36
	Transitional-e	CD34- CD10+		6.73	12.95	0.00	50.80		0.07	0.13	0.00	0.62
	Transitional-d	CD21- CD38+		22.53	20.80	0.35	90.10		11.34	17.86	0.00	96.90
	Transitional-a	CD10+ CD38+		9.64	15.03	0.00	71.10		0.68	1.13	0.00	4.81
	Anergic	CD21(−/low)CD38-		24.21	26.20	2.35	97.20		21.63	16.61	0.60	95.50

*DLBCL* Diffuse large B cell lymphoma

**Fig. 2:**
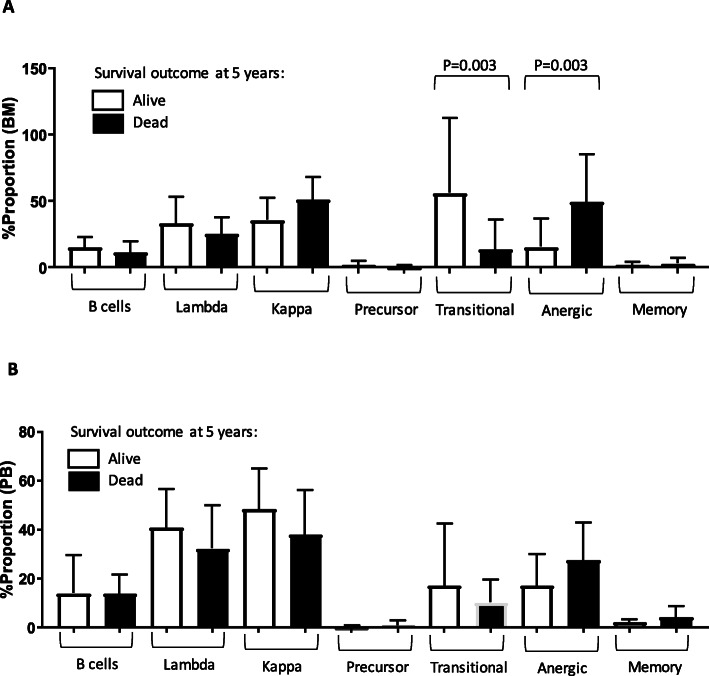
Low proportions of transitional B cells and high proportions of anergic B cells in the BM are significantly associated with negative survival outcome at 5 years. Proportions of B cells and subpopulations in **a.** BM and **b.** PB samples of 75 DLBCL patients at diagnosis were quantified using multicolor flow cytometry. Survival outcome at 5-years (yes/no) data was available on *N* = 24 BM and N = 21 PB blood samples as plotted above. Comparison was made using exact non-parametric Mann-Whitney U test. For this analysis, *p* < .01 was taken to be significant. Graph represents mean ± SD

The proportion of total B cells and various subpopulations was compared using Mann – Whitney U test between patients where we chose 5 years after diagnosis as an adequate timepoint to assess survival as a binary outcome (yes/ no). For this analysis, we excluded patient samples where follow up data was less than 5 years. Of the samples analyzed, which included PB (*N* = 21) and BM (*N* = 24), there were no significant differences in any B cell subpopulations in the PB; however, the proportion of anergic B cells (*p* = 0.003) and transitional B cells (p = 0.003) were significantly different in the BM at the 5-year survival outcome point (Fig. 2a and b). For this analysis, *p*-value of <.01 was taken to be significant.

**Fig. 3:**
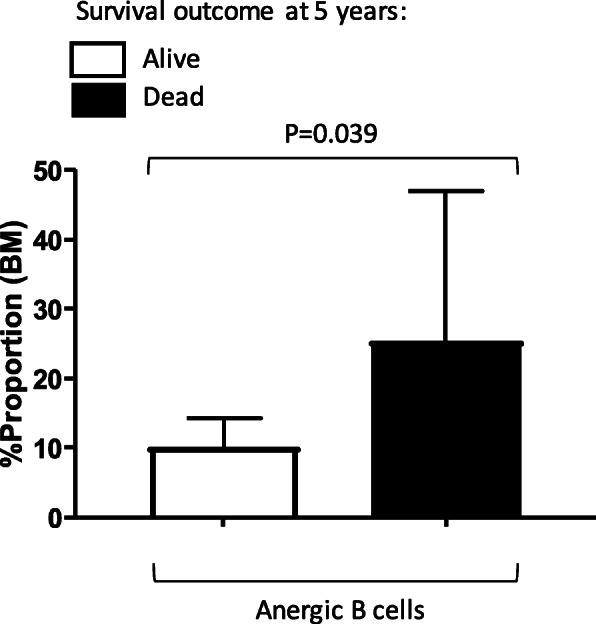
High proportion of Anergic B cells in the BM is significantly associated with negative survival outcome at 5 years independent of BM involvement. Proportions of anergic B cells in the BM of DLBCL patients at diagnosis were quantified using multicolor flow cytometry. Survival outcome at 5-years (yes/no) data was available on *N* = 24 BM samples but presence of clonal cells was detected in *N* = 9 of BM cases which were excluded in this analysis. The presence of these cells was determined and recorded previously as cells having an abnormal kappa: lambda light chain ratio with normal expression defined as 3:2 on flow cytometry. Comparison was made using exact non-parametric Mann-Whitney U test. For this analysis, *p* < .05 was taken to be significant. Graph represents mean ± SD

We repeated the analysis as described above for transitional and anergic B cells looking at 5-year survival status after excluding 9 out of 24 BM samples that had marrow involvement recorded where clonal lymphoma cells were detected in the BM. The phenotype of clonal lymphoma cells was determined and recorded previously as cells having an abnormal kappa: lambda light chain ratio with normal expression defined as 3:2 on flow cytometry. This exclusion analysis was done to evaluate the importance of normal BM B cells in defining survival in DLBCL and exclude the possibility of BM clonal cells contributing to the survival difference. We found that the proportion of anergic B cells in the BM were still significantly different (*p* = 0.039), i.e. it was higher in patients who died at 5 year following diagnosis compared to those that were still alive, and when there was no disease detected in the BM (Fig. 3). Moreover, the phenotype of the clonal cells in the BM as evaluated by flow cytometry was found to be highly heterogenous and did not always associate with an “anergic” feature, i.e. CD21−/low expression (unpublished data). Also, the proportion of transitional B cells were no longer significant in this analysis after BM exclusion (data not shown).

### High proportion of anergic B cells in the BM is associated with significantly shorter OS

**Fig. 4:**
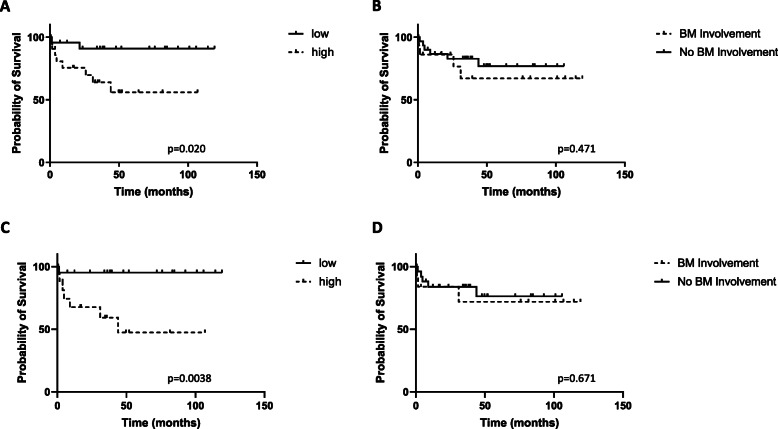
High proportion of anergic B cells in the BM is associated with significantly shorter overall survival. **a.** Proportions of anergic B cells in the BM (*N* = 47) of DLBCL patients at diagnosis were quantified using multicolor flow cytometry to look at its impact on overall survival. For this, the BM data was divided into anergic B cell “high” and “low” groups using a median split approach where proportions > 13.9% was “high” and ≤ 13.9% was “low”. **b.** BM involvement (yes/no) in the same cohort of patients was also determined to look at how it compares with overall survival. Above analysis was repeated on DLBCL not otherwise specified (*N* = 40) after excluding primary central nervous system DLBCL (N = 2) and transformed disease (*N* = 5) for **c.** “high” and “low” anergic B cell BM marrow proportions and **d.** BM involvement (yes/no). BM involvement was determined and recorded previously as detection of cells having an abnormal kappa: lambda light chain ratio with normal expression defined as 3:2 on flow cytometry. Overall survival was measured as the number of months from the date of diagnosis to the date of death or date of last follow-up (censored). For this Kaplan Meier analysis, p < .05 was taken to be significant

The entire cohort of DLBCL patients with BM data (*N* = 47) was divided into anergic B cell “high” and “low” groups using a median split approach to measure differences in OS. Anergic B cell proportions > 13.9% was “high” and ≤ 13.9% was “low”. Patients with a “high” proportion of anergic B cells in the BM had a significantly shorter OS than those with a “low” proportion (*p* = 0.020) (Fig. 4a). Furthermore, when we evaluated the same cohort of patients to look at the association between OS and BM involvement (*N* = 47), it was not found to be significant (*p* = 0.470) (Fig. 4b). Next, we excluded primary central nervous system (*N* = 2) and transformed (*N* = 5) DLBCL samples from this pool and evaluated DLBCL not otherwise specified (NOS) (*N* = 40) samples for analysis as above. We found that “high” proportion of anergic B cells in the BM was associated with a significantly poorer survival (*p* = 0.0038) (Fig. 4c) when compared to the “low” group whereas presence or absence of BM involvement did not yield significant difference in survival (*p* = 0.671) (Fig. 4d).

The proportion of anergic B cells in the BM was not associated with BM involvement (*p* = 0.22), confirming that these B cells were part of the microenvironment (data not shown). Interestingly, the proportion of anergic B cells did not associate with refractory or relapse disease, both in the BM (*p* = .264) and the PB (*p* = .605) samples. “High” anergic B cell group was significantly associated with an increase in median age (67 vs. 56, *p* = 0.013) and high category of performance status ECOG score (*p* = 0.017) when compared with the “low” group. The type of treatment received between the two groups were not statistically significant. Interestingly, fisher’s exact test revealed that “low” anergic B cells in the BM associated with GCB cell phenotype (*p* = 0.0345) that has been established to have superior prognosis, when compared to ABC phenotype, in DLBCL.

### Poor prognosis associated with high proportions of anergic B cells in the BM is independent of IPI in DLBCL

When OS as a dependent variable was utilized in a Cox regression analysis, only high proportion of anergic B cells in the BM (*p* = 0.010) and R-IPI score (*p* = 0.007) was able to predict OS survival independently (*N* = 47) (Table 2). Other B cell populations in the BM or PB (*N* = 54), including transitional B cells in the BM was not significant in this model (data not shown). Similarly, BM involvement was also not significant (Table 2).

**Table 2:** Cox regression analysis shows that high proportion of anergic B cells in the BM (N = 47) and R-IPI score predicts overall survival independently. The same analysis for BM involvement is not significant. For this analysis, *p*-value of <.05 was taken to be significant

	Sig.	Hazard Ratio	95.0% CI for Hazard ratio	
			Lower	Upper
R-IPI Score	0.007	2.18	1.23	3.85
% Anergic B cells BM	0.010	1.04	1.01	1.08
BM involvement	0.160	0.20	0.02	1.90

*R-IPI* Revised International Prognostic Index, *BM* Bone marrow

## Discussion

We report the first attempt to characterize the prognostic impact of various B cell subpopulations in the BM and PB of DLBCL patients. Initial analysis has revealed that low proportions of transitional (CD10+/CD34-, CD38+/CD21- and CD10+/CD38+) and high proportions of anergic (CD21(−/low)/CD38-) B cells in the BM were negatively associated with 5-year survival outcome.

High proportion of anergic B cells in the BM was significantly associated with poor survival outcomes at 5 years independent of BM involvement at the time of diagnosis, i.e. the presence of clonal cells in the marrow. Further analysis revealed that high proportion of anergic B cells was associated with a significantly shorter OS. When we evaluated the same cohort of patients to predict overall survival with BM involvement, it was not significant. Finally, with this cohort, high proportion of anergic B cells was an independent prognostic marker for poor OS (*p* = 0.010) independent of R-IPI (*p* = 0.007).

Anergic B cells serve to avoid autoimmune reactions by being functionally limited and unresponsive to antigen stimulation [40–42]. They have been primarily identified as CD21−/low cells by flow cytometry [43]. Multiple other markers and functional studies have been used to define the antigen variability of anergic B cells in both healthy individuals and in disease states [44]. Some recent studies have shown that the phenotype of CD21(−/low) cells is not purely anergic; these cells have also been identified as memory B cells [45]. In other studies, the CD21(−/low) cells defined as anergic also express low or absent CD38 antigen [37–39]. Our anergic B cell immunophenotyping best fit this profile; however, we acknowledge that further studies must be done to functionally characterize these cells as being anergic in DLBCL, preferably in a larger cohort. Irrespective of other markers, CD21(−/low) expression is mostly associated with an inability of B cells to mount an immune response [46] and in our study CD21(−/low)/CD38- expression is significantly associated with clinical outcome in DLBCL.

The ability of malignant cells to evade immune response is a known hallmark feature of carcinogenesis [47]. The interaction of lymphoma cells with the immune cells in the TME is known to promote antitumor activity that keeps the host in an immunosuppressive state [13, 48, 49]. Therefore, high proportions of anergic B cells in the BM may serve to define long term “immunosuppressive” TME. It is possible that this is not entirely evident by examining cells of the PB. This phenomenon if further validated could be a strong rationale for performing BM biopsy in all DLBCL patients to examine the presence of CD21(−/low) cells.

High numbers of anergic B cells have previously been described for chronic lymphocytic leukemia (CLL) where the presence of these cells allow the survival of leukemic lymphocytes causing aggressive disease [50, 51]. In CLL, the anergic B cells have been described to be clonal in nature [50, 51]. In our study, we found that the association of high anergic B cells in the BM with poor survival was independent of presence of clonal cells in the marrow, suggesting that these B cells could be part of the normal immune population.

It is also interesting to note that the proportion of high anergic B cells in the BM and PB did not associate with refractory or relapse disease, suggesting it does not lead to a “chemoresistance” phenotype. Having said that, targeting these cells could have important implications for immunotherapy, in fact reversal of anergic phenotype in CLL has been proposed to be beneficial as a therapy for the disease [50]. Finally, the prognostic value of using the proportion of anergic B cells in the BM as a predictor of poor survival needs to be further evaluated in a larger cohort of DLBCL patients. We will be expanding the analysis on a larger cohort to validate our results.

## Conclusions

High proportion of anergic B cells in the BM characterized by CD21(−/low)/CD38- expression is an independent marker of poor prognosis in DLBCL. If validated in a larger study, testing of these cells in the BM should be considered as part of prognostic profiling. Future studies should also functionally characterize these cells and explore its potential as a target for immunotherapy.

## Data Availability

The datasets generated and/or analyzed during the current study are available from the corresponding author on reasonable request.

## Abbreviations

DLBCL: Diffuse large B cell lymphoma
R-IPI: Revised International Prognostic Index
GCB: Germinal Center B cell
ACB: Activated B cell
OS: Overall survival
TME: Tumour microenvironment
PB: Peripheral Blood
BM: Bone marrow
MNC: Mononuclear cells
CLL: Chronic lymphocytic leukemia
